# Ultrasound Identification of Retrobulbar Hematomas by Emergency Physicians in a Cadaveric Model

**DOI:** 10.5811/westjem.2020.1.45081

**Published:** 2020-04-13

**Authors:** Edward Carlin, Alexa Palmieri, Tanya Bajaj, Mathew Nelson

**Affiliations:** * North Shore University Hospital, Department of Emergency Medicine, Manhasset, New York; † North Shore University Hospital, Department of Emergency Medicine, Division of Emergency Ultrasound, Manhasset, New York

## Abstract

**Introduction:**

Retrobulbar hemorrhage (RBH) is a rare complication of facial trauma that can lead to dangerous orbital compartment pressures and must be rapidly recognized to prevent permanent vision loss. Point-of-care ultrasound (POCUS) offers a rapid modality for evaluating a wide variety of ocular pathologies, and prior case reports demonstrate the ability of clinicians to recognize RBH using ultrasound. This study aimed to assess the ability of clinicians at various stages of training to identify a RBH using POCUS in a cadaveric model. Clinicians also were assessed for self-reported comfort using ultrasound for ocular pathology before and after the study.

**Methods:**

Participants included 17 physicians who evaluated 10 eyes (from five cadavers) that were independently randomized to have either a modeled RBH or no hemorrhage. Participants’ final diagnosis of each eye was recorded (RBH present or not), and participants also completed pre- and post-activity surveys.

**Results:**

The overall sensitivity and specificity to correctly diagnose retrobulbar fluid was 87% and 88%, respectively. Sensitivity and specificity were higher after excluding clinicians in their early phase of training. Additionally, self-reported comfort level with ocular ultrasound was significantly improved by this activity.

**Conclusion:**

Emergency physicians at a variety of training levels can correctly identify a cadaveric model of retrobulbar hemorrhage. Use of this cadaveric model can improve exposure of clinicians to the appearance of a rare but vision-threatening ocular pathology such as RBH.

## INTRODUCTION

Retrobulbar hemorrhage (RBH) is a rare complication of facial trauma; however, it must be rapidly recognized to prevent permanent vision loss from increased orbital compartment pressures.^1^–^3^ Typically, emergency physicians must identify this entity based upon the patient’s clinical presentation, or on delayed imaging with computed tomography (CT).^4^ Uncertainty during the clinical examination and delays in obtaining a CT may lead to further progression of RBH and vision loss. Point-of-care ultrasound (POCUS) represents a rapid, repeatable method of imaging ocular pathology, without need for transport out of the emergency department (ED), and can be done concurrent with other traumatic injury management.^5^ RBH has been identified using bedside ocular ultrasound in several case reports; however, no systematic studies of this condition exist, likely due to the rare nature of this injury.^5^

Cadaver models represent a useful way to simulate rare pathologies for both research and educational purposes.^6^,^7^ This is important not only for advancing our understanding of certain disease processes, but also to allow clinicians to become exposed to pathology not seen in day-to-day practice.^6^–^8^ Previous work has shown the practicality of reproducing a variety of ocular pathologies for visualization under ultrasound, including RBH.^8^ In this pilot study, we demonstrate the feasibility of simulating RBH in cadaver models for imaging with POCUS. As a primary endpoint, we analyzed the ability of residents, emergency ultrasound fellows, and ED faculty physicians to accurately diagnose the presence of retrobulbar fluid in our models. As a secondary endpoint, we surveyed the participants about their comfort level with and likelihood to use ocular ultrasound before and after the activity.

## METHODS

**Figure 1:**
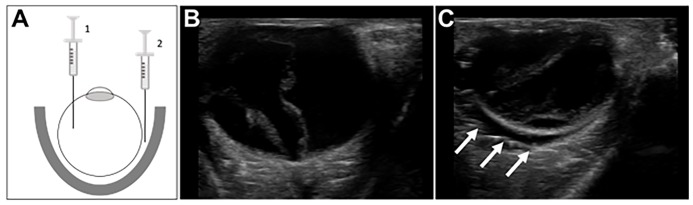
Preparation and representative images of the retrobulbar hemorrage model. (A) Fresh frozen cadavers were first prepared by injecting 5–10 cubic centimeters (cc) of normal saline into the posterior chamber (1) to recreate normal anatomic arrangement of the globe. Subsequently, 10–20 cc of a mixture of gel and saline was instilled within the retrobulbar area (2) by carefully inserting a 20-gauge needle between the globe and lateral aspect of the orbit. (B–C) Examples of images obtained from the cadaveric models. Panel B shows a “normal” eye, and Panel C shows an eye following placement of retrobulbar fluid (arrows). In both cases there are hyperechoic linear foci within the posterior body, which was seen in all cadavers and appears consistent with a retinal detachment.

This study was accomplished in the Bioskills laboratory of Northwell Health Systems, using fresh frozen cadavers and in compliance with department policies and institutional review board standards. To prepare the eyes for analysis, 5–10 milliliters (mL) of normal saline was injected into the posterior chamber using a 22-gauge needle to restore the normal anatomic shape (as cadaveric eyes become desiccated). Five cadavers were available for this study, and each eye (10 in total) was independently randomized to be either normal or have RBH. For the eyes randomized to the RBH group, 10–20 mL of a normal saline and gel mixture (1:1) was instilled posterior to the eye under ultrasound guidance (Figure 1A). Participants included residents of various levels of training (postgraduate year [PGY]-1 to PGY-3), two ultrasound fellows, and five attendings (Table 1).

Each participant was given a pre-activity survey and a brief instructional introduction on the use of POCUS for ocular pathology and the appearance of RBH on ultrasound. During the introduction, participants were given a 15-minute didactic presentation summarizing techniques for ocular POCUS, representative normal images, and examples of RBH. Each participant had the opportunity to use either a SonoSite M-Turbo ultrasound (Bothell, WA) equipped with a linear 10 megahertz array, or a Philips Lumify L12-4 linear array (Amsterdam, The Netherlands) (attached to an Android-based LCD screen) on each cadaveric eye. Participants then recorded the presence or absence of a RBH for each eye. Participants completed a post-activity survey regarding their comfort level with ocular ultrasound and likelihood to use in clinical practice. Following completion of the study, each participant’s surveys and score sheet were given a unique randomized number without additional identifying information.

Results of participants’ evaluation of each eye were recorded and analyzed for sensitivity and specificity, and further analyzed by subgroups according to level of training. Additionally, survey responses for all groups were recorded and a comparison was made for each question regarding comfort level and likelihood to use POCUS for evaluation of ocular pathology.

## RESULTS

In this pilot study, a total of 17 participants evaluated 10 eyes (five cadavers) prepared for this investigation (five eyes with “normal” anatomy and five eyes modeled to have RBH). Figure 1 shows representative images obtained during this activity. Figure 1B demonstrates an eye after instillation of saline to approximate normal anatomy; it should be noted that there is evidence of retinal detachment, which was incidentally seen on most eyes and likely represents damage sustained post-mortem or from frozen storage. Figure 1C demonstrates an eye with an anechoic collection posterior to the globe, representing the modeled RBH.

**Population Health Research Capsule**

What do we already know about this issue?

Retrobulbar hemorrhage, a rare complication of facial trauma, must be rapidly recognized to prevent permanent vision loss.

What was the research question?

This study assessed if clinicians could identify a cadaver model of retrobulbar hemorrhage using point-of-care ultrasound.

What was the major finding of the study?

Emergency physicians could accurately identify the ultrasound findings of a retrobulbar hemorrhage in a cadaver model.

How does this improve population health?

This model can improve the familiarity of clinicians to the ultrasound findings seen in patients with retrobulbar hemorrhage.

The participants were able to distinguish those eyes with a modeled RBH from “normal” anatomy, with an overall sensitivity and specificity of 0.87 and 0.88, respectively (Table 1). This increased among participants at PGY-3 training level or higher. PGY-3 participants had a sensitivity and specificity of 1.00 and 0.93, respectively, while two ultrasound fellows had 1.00 and 1.00. Paradoxically, among participating faculty members the sensitivity and specificity decreased to 0.92 and 0.92. For pooled results for participants at PGY-3 or higher, the sensitivity was 0.96 and specificity was 0.94.

The participants reported their comfort level with using physical exam and POCUS for evaluating ocular pathology before and after the exercise, using a five-point Likert scale. Both saw statistically significant increases in comfort level after completion, based upon an increased, self-reported comfort level.

## DISCUSSION

RBH is a rare but potentially devastating injury that must be rapidly recognized to treat effectively; however, concomitant craniofacial injuries can affect an examiner’s ability to recognize signs of RBH on physical exam. Additionally, the time associated with obtaining and reading CT images can lead to dangerous delays in diagnosis. POCUS can be used to rapidly screen for RBH at the patient’s bedside and without the patient having to leave the department for imaging. Previous case reports have documented the use of POCUS to identify RBH; however, the rarity of this pathology has made more systematic research difficult.

Our pilot study indicates that human cadaver models can be used to effectively train emergency medicine practitioners to recognize sonographic signs of a RBH. After the completion of a focused training session, the participants successfully identified a RBH on ultrasound with a sensitivity and specificity that increased with the level of postgraduate training. We noted a sensitivity of 0.96 and a specificity of 0.94 with recognizing RBH in those PGY-3 level of training or higher. Physicians currently enrolled in the ultrasound fellowship who participated in this study had a sensitivity and specificity of 100%, suggesting that more advanced training can further increase the diagnostic utility of ocular ultrasound. Attending scores decreased slightly to 0.92 (sensitivity and specificity), likely reflecting higher proficiency among ultrasound fellows with more recent, advanced training.

Using our pre- and post-study questionnaires, our study showed a significant increase in participants’ comfort level in using POCUS to diagnose RBH. Physician likelihood to use ocular ultrasound was unchanged from the activity; however, given that the ranking was initially high (4 out of 5), any increase in likelihood-to-use may not be measurable with the low sample size obtained. Based upon these findings, fresh frozen cadavers can provide an accurate educational simulation of RBH on ultrasound.

## LIMITATIONS

Our study had several limitations. The sample size was small and lacked the ability to test the same number of residents and attendings per PGY level. Although using fresh frozen cadavers provided simulation of true pathology, the lack of eye movement seen in live patients may have allowed for easier detection of RBH. The cadaver models did not represent several other aspects of real-life diagnosis, such as patient discomfort, uncooperative patients, or concomitant traumatic injuries (including orbital or facial trauma, which may prevent visual inspection of the eye or measurement of intraocular pressure). Additionally, this cadaver model contained other ocular pathology on ultrasound, such as the appearance of retinal detachments (likely resulting from cadaver preparation). This study was also limited to the evaluation of RBH greater than 10 cubic centimeters in volume, and there is likely a decrease in sensitivity and specificity with smaller volumes. Future studies could assess the accuracy of identifying different retrobulbar volumes. Despite these limitations, this approach represents a feasible method for reliably representing a rare pathology for physician education.

## CONCLUSION

Emergency physicians can use POCUS to correctly identify a cadaveric model of RBH. Higher levels of training seem to correlate with improved accuracy in diagnosing this condition. Use of this model can improve clinicians’ exposure to the appearance of rare, vision-threatening ocular pathology, such as RBH. More study will be needed to assess the accuracy of POCUS for identifying retrobulbar hemorrhage in live patients.

## Figures and Tables

**Table 1:** Sensitivity and specificity for diagnosing retrobulbar hemorrhage on point-of-care ultrasound on POCUS by physician training level.

Level of training	Number of participants	Sensitivity	Specificity
PGY-1	4	0.88	0.67
PGY-2	3	0.80	0.87
PGY-3	3	1.00	0.93
Fellow	2	1.00	1.00
Attending	5	0.92	0.92

Overall	17	0.87	0.88
*PGY-3+*	*10*	*0.96*	*0.94*

*PGY*, postgraduate year.
